# Supplementary material for: Predicting Regions of Local Recurrence in Glioblastomas Using Voxel-Based Radiomic Features of Multiparametric Postoperative MRI
Source: Cancers (Basel). 2023 Mar 22;15(6):1894. doi: 10.3390/cancers15061894 (PMC10047582; doi:10.3390/cancers15061894)
Supplement: Supplementary file 1 [file cancers-15-01894-s001.zip › cancers-2257409-supplementary.pdf]

Supplementary Material

Table of contents

Table S1. MRI acquisition parameters used by participating institutions ……… Page 2
Table S2. Pyradiomics settings used for voxel-wise feature extraction………… Page 3

Table S1. MRI acquisition parameters used by participating institutions					
**MRI Sequence**	**Río Hortega University Hospital, Valladolid, Spain**	**12 de Octubre University Hospital, Madrid, Spain**	**St. Olavs University Hospital, Trondheim Norway**	**Case Western Reserve University, Cleveland, USA**	**University of Pennsylvania, Philadelphia, USA**
Manufacturer, model and Field strength	General Electric, Signa HDxT, 1.5 T	General Electric, Signa Premier, 3 T	Siemens, Skyra, 3 T	Siemens, Avanto, 1.5 T	Siemens, TrioTim, 3 T
T1ce	TR/TE/FA, 7.98 ms/2.57 ms/12º; 3D; GRE; FOV, 220 x 220 mm; matrix, 512 x 512; slice thickness, 1 mm	TR/TE/FA, 6.82 ms/2.16 ms/12º; 3D; FSPGRE; FOV, 320 x 320 mm; matrix, 512 x 512; slice thickness, 1 mm	TR/TE/FA, 2000 ms/2.96 ms/8º; 3D; MPRAGRE; FOV, 256 x 256 mm; matrix, 256x 256; slice thickness, 1 mm	TR/TE, 589 ms/12 ms; 2D; TSE; FOV, 256 x 256 mm; matrix, 256x 179; slice thickness, 5 mm	TR/TE, 1760 ms/3.11 ms; 3D; GRE; FOV, 256 x 187 mm; matrix, 256x 192; slice thickness, 1 mm
T1w	TR/TE, 580 ms/7.56 ms; 2D; FSE; FOV, 220 x 220 mm; matrix, 512 x 512; slice thickness, 5 mm	TR/TE/FA, 6.85 ms/2.12 ms/12º; 3D; FSPGRE; FOV, 320 x 320 mm; matrix, 512 x 512; slice thickness, 5 mm	TR/TE/FA, 2000 ms/2.96 ms/8º; 3D; MPRAGRE; FOV, 256 x 256 mm; matrix, 256 x 256; slice thickness, 1 mm	TR/TE, 529 ms/12 ms; 2D; TSE; FOV, 256 x 224 mm; matrix, 256 x 224; slice thickness, 5 mm	TR/TE, 1760 ms/3.11 ms; 3D; GRE; FOV, 187 x 250 mm; matrix, 256 x 192; slice thickness, 1 mm
T2w	TR/TE, 5220 ms/96.12 ms; 2D; FRSE; FOV, 220 x 220 mm; matrix, 512 x 512; slice thickness, 5 mm.	TR/TE, 4322 ms/124.25 ms; 2D; FSE; FOV, 256 x 256 mm; matrix, 512 x 512; slice thickness, 4 mm	TR/TE, 4200 ms/88 ms; 2D; TSE; FOV, 256 x 256 mm; matrix, 512 x 359; slice thickness, 3 mm	TR/TE, 5000 ms/91 ms; 2D; TSE; FOV, 256 x 224 mm; matrix, 256 x 224; slice thickness, 5 mm	TR/TE, 5340 ms/85 ms; 2D; TSE; FOV, 240 x 195 mm; matrix, 256 x 208; slice thickness, 3 mm
FLAIR	TR/TE, 8002 ms/135.07 ms; 2D; FSE; FOV, 220 x 220 mm; matrix, 512 x 512; slice thickness, 4 mm	TR/TE, 9350 ms/142.56 ms; 2D; FSE; FOV, 320 x 224 mm; matrix, 512 x 512; slice thickness, 4 mm	TR/TE, 9000 ms/108 ms; 3D; TSE; FOV, 256 x 256mm; matrix, 256 x 256; slice thickness, 1 mm	TR/TE, 9000 ms/109 ms; 2D; TSE; FOV, 256 x 208 mm; matrix, 256 x 208; slice thickness, 5 mm	TR/TE, 9420 ms/141 ms; 2D; TSE; FOV, 240 x 180 mm; matrix, 256 x 192; slice thickness, 3 mm
DWI	TR/TE, 8000 ms/111.7 ms; FOV, 256 x 256 mm; matrix, 128 x 160; slice thickness, 5 mm; b-values, 0 and 1000 s/mm2	TR/TE, 3780 ms/57.2 ms; FOV, 256 x 256 mm; matrix, 160 x 160; slice thickness, 3.5 mm; b-values, 0 and 1000 s/mm2	TR/TE, 8130 ms/64 ms; FOV, 160 x 160 mm; matrix, 160 x 160; slice thickness, 3 mm; b-values, 0 and 1000 s/mm2	TR/TE, 4700 ms/89 ms; FOV, 160 x 160 mm; matrix, 160 x 160; slice thickness, 5 mm; b-values, 0 and 1000 s/mm2	TR/TE, 5000 ms/86 ms; FOV, 192 x 192 mm; matrix, 192 x 192; slice thickness, 3 mm; b-values, 0 and 1000 s/mm2

T1ce = contrast-enhanced T1w, T2w= T2-weighted image, FLAIR = Fluid-attenuated inversion recovery, DWI = diffusion weighted image, TR = repetition time, TE= echo time, FOV = field of view. GRE = gradient echo. TSE = turbo spin echo. FSE= fast spin echo. FRFSE= fast recovery fast spin echo. FSPGR= Fast Spoiled Gradient Echo. MPRAGE= Magnetization Prepared RApid Gradient Echo

**Table S2. Pyradiomics settings used for voxel-wise feature extraction**	
Image Type Filter	Original: {}<br>Wavelet: {}<br>LBP3D:<br>binWidth: 1.0<br>LoG:<br>sigma: [1.0, 3.0, 5.0]
Feature Class	First order:<br>- Energy<br>- Total energy<br>- Entropy<br>- Minimum<br>- 10th percentile<br>- 90th percentile<br>- Maximum<br>- Mean<br>- Median<br>- Interquartile Range<br>- Range<br>- Mean Absolute Deviation<br>- Root mean squared<br>- Standard deviation<br>- Skewness<br>- Kurtosis<br>- Variance<br>- Uniformity<br>Gray Level Co-occurrence Matrix (GLCM):<br>- Autocorrelation<br>- Joint Average<br>- Cluster Prominence<br>- Cluster Shade<br>- Cluster Tendency<br>- Contrast<br>- Correlation<br>- Difference Average<br>- Difference Entropy<br>- Difference Variance<br>- Joint Energy<br>- Joint Entropy<br>- Informational Measure of Correlation (IMC) 1<br>- Informational Measure of Correlation (IMC) 2<br>- Inverse Difference Moment (IDM)<br>- Inverse Difference Moment Normalized (IDMN)<br>- Inverse Difference<br>- Inverse Difference Normalized<br>- Inverse Variance<br>- Maximum Probability<br>- Sum Entropy<br>- Sum Squares<br>Gray Level Run Length Matrix (GLRLM):<br>- Short Run Emphasis (SRE)<br>- Long Run Emphasis (LRE)<br>- Gray Level Non-Uniformity (GLN)<br>- Gray Level Non-Uniformity Normalized (GLNN)<br>- Run Length Non-Uniformity (RLN)<br>- Run Length Non-Uniformity Normalized (RLNN)<br>- Run Percentage (RP)<br>- Gray Level Variance (GLV)<br>- Run Variance (RV)<br>- Run Entropy (RE)<br>- Low Gray Level Run Emphasis (LGLRE)<br>- High Gray Level Run Emphasis (HGLRE)<br>- Short Run Low Gray Level Emphasis (SRLGLE)<br>- Short Run High Gray Level Emphasis (SRHGLE)<br>- Long Run Low Gray Level Emphasis (LRLGLE)<br>- Long Run High Gray Level Emphasis (LRHGLE)<br><br>Gray Level Size Zone Matrix (GLSZM):<br>- Small Area Emphasis (SAE)<br>- Large Area Emphasis (LAE)<br>- Gray Level Non-Uniformity (GLN)<br>- Gray Level Non-Uniformity Normalized (GLNN)<br>- Size-Zone Non-Uniformity (SZN)<br>- Size-Zone Non-Uniformity Normalized (SZNN)

	- Zone Percentage (ZP)<br>- Gray Level Variance (GLV)<br>- Zone Variance (ZV)<br>- Zone Entropy (ZE)<br>- Low Gray Level Zone Emphasis (LGLZE)<br>- High Gray Level Zone Emphasis (HGLZE)<br>- Small Area Low Gray Level Emphasis (SALGLE)<br>- Small Area High Gray Level Emphasis (SAHGLE)<br>- Large Area Low Gray Level Emphasis (LALGLE)<br>- Large Area High Gray Level Emphasis (LAHGLE)<br><br>Neighbouring Gray Tone Difference Matrix (NGTDM):<br>- Coarseness<br>- Contrast<br>- Busyness<br>- Complexity<br>- Strength<br><br>Gray Level Dependence Matrix (GLDM):<br>- Small Dependence Emphasis (SDE)<br>- Large Dependence Emphasis (LDE)<br>- Gray Level Non-Uniformity (GLN)<br>- Dependence Non-Uniformity (DN)<br>- Dependence Non-Uniformity Normalized (DNN)<br>- Gray Level Variance (GLV)<br>- Dependence Variance (DV)<br>- Dependence Entropy (DE)<br>- Low Gray Level Emphasis (LGLE)<br>- High Gray Level Emphasis (HGLE)<br>- Small Dependence Low Gray Level Emphasis (SDLGLE)<br>- Small Dependence High Gray Level Emphasis (SDHGLE)<br>- Large Dependence Low Gray Level Emphasis (LDLGLE)<br>- Large Dependence High Gray Level Emphasis (LDHGLE)
Settings:	normalize: true<br>normalizeScale: 100<br>binWidth: 25<br>interpolator: 'sitkBSpline'<br>resampledPixelSpacing: [1, 1, 1]<br>correctMask: true<br>voxelArrayShift: 300<br>force2D: true<br>force2Ddimension: 0
Voxel settings	kernelRadius: 1 [3 x 3 x 3]<br>maskedKernel: true<br>voxelBatch: 10000
